# Shifting Attention and Response Time Performance in Adolescents: Effects of External and Internal Focus

**DOI:** 10.3390/sports14060225

**Published:** 2026-05-29

**Authors:** Fábio Flôres, Priscila Cardozo, Denise Soares, Ricardo Drews

**Affiliations:** 1 Universidade de Évora, Centro de Investigação em Educação e Psicologia (CIEP), 7004-516 Évora, Portugal; fabio.flores@uevora.pt; 2 Universidade de Évora, Comprehensive Health Research Centre (CHRC), 7004-516 Évora, Portugal; 3 Universidade de Évora, Escola de Ciências Sociais, 7004-516 Évora, Portugal; 4 Escola Superior de Educação Física, Universidade Federal de Pelotas, Pelotas 96010-610, Brazil; priscilacardozo88@gmail.com; 5 Liberal Arts Department, American University of the Middle East, Egaila 54200, Kuwait; 6 Centro de Educação Física e Desportos, Universidade Federal de Santa Maria, Santa Maria 97105-900, Brazil; ricardo.drews@gmail.com

**Keywords:** attention, response time, psychomotor performance, school, sports

## Abstract

Aim: To examine the effects of attentional focus on adolescents’ response time performance and investigate whether sports participation moderates this effect. Methods: Fifty-eight adolescents (16.46 years) were randomly assigned to one of three groups: external focus of attention (EF), internal focus of attention (IF), or control. Participants performed a response-time task using a visual stimulus. A 3 × 2 factorial ANOVA was conducted, with attentional focus group (external, internal, control) and sports participation (yes, no) as between-subjects factors. Results: A significant main effect of attentional focus was found, with the EF group outperforming the IF and control groups. Sports participation alone was not a significant factor, and the interaction between attentional focus and sports participation was not statistically significant. However, exploratory analyses suggested a possible tendency for adolescents engaged in sports practice to respond more favorably to an EF strategy, though these findings should be interpreted with caution. No significant differences were observed among non-sports participants. Conclusion: These findings reinforce the benefits of an external attentional focus for adolescent response-time performance. Although exploratory, the results suggest that adolescents engaged in sports practice may respond more favorably to EF, highlighting the potential relevance of reconsidering commonly used IF instructions in youth sports and educational contexts.

## 1. Introduction

Sports coaches and physical educators regularly use a variety of strategies to improve motor skills among their athletes and students. Verbal instructions and feedback are the most common forms of information about the practice, providing participants with essential data about the movement. Generally, those professionals often direct participant attention to a specific body part during movement, known as the internal focus of attention (IF). In fact, professionals predominantly used IF during training and classes, with a slight variation during practice and competition [1]. However, various studies have challenged this practice [2,3,4]. The literature suggests that learning and performance can be improved when participants’ attention is directed to the effects of their movements on the environment, known as the external focus of attention (EF) [5,6]. Recent investigations have also shown that the focus of attention influences motor behavior during everyday activities and sports tasks [7]. Those results are supported by the Constrained Action Hypothesis, which suggests that EF could facilitate automatic movement control, whereas an IF would induce conscious movement control, thereby disrupting the motor system and consequently impairing performance [8,9,10]. Therefore, when adopting an EF, the performer is more likely to exhibit a fast process governed by lower-level movement control, thereby facilitating the achievement of the desired outcome [11].

In recent years, investigations have shown that the EF enhances motor skill acquisition and performance compared with the IF, regardless of age, sport, task, sex, or condition [12,13,14,15,16,17,18]. When coaches or teachers direct performers’ attention toward specific movement-related cues, attentional focus may influence how motor actions are processed and executed [19]. For example, a soccer player may focus either on the intended movement outcome (EF) or on their own body movements (IF) when manipulating the ball. Previous research consistently suggests that EF enhances motor learning and performance across different tasks and populations [7,20,21], whereas IF may constrain movement efficiency [22]. Thus, how the student/athlete uses the information to guide their actions may contribute to or limit their motor performance.

The general literature has consistently shown the advantages of adopting an EF, primarily in adults [13,23,24] and children [12,19,25]. Since Wulf et al. [6], much research has suggested that EF can improve motor learning and performance in these populations [12,13,14,26,27,28]. Nevertheless, there remain limited investigations into adolescent performance [19,29,30]. Adolescence represents a critical developmental stage characterized by ongoing maturation of cognitive, perceptual–motor, and neuromuscular systems, all of which may influence attentional control and motor performance. During this period, improvements in executive functioning, information processing, motor coordination, and decision-making [31,32] may change how attentional focus strategies are processed and applied during motor tasks. Therefore, investigating attentional focus effects in adolescents may provide important insights into perceptual–motor functioning during a stage of substantial developmental adaptation. Saemi et al. [19,29] suggested that EF of attention can enhance the performance of experienced adolescent swimmers, but not of beginners. Schwab et al. [29] showed that the EF group improved more than the IF group during a free-kick task. In another investigation, Marchant et al. [30] showed that the EF can perform better during a standing long jump task. In addition, recent systematic reviews have indicated that instructions/feedback can influence performance when inducing practitioners’ attention [20,21].

As far as we know, there is still limited evidence in the literature on the effects of different attentional foci on adolescent performance, particularly with respect to response time; thus, further research is needed to explore the impact of different attentional foci in this population. This gap limits our understanding of how different attentional foci can influence a highly demanding information-processing task, such as response time, which is considered an important perceptual–motor performance indicator as it reflects the efficiency of attentional processing, stimulus detection, motor preparation, and movement execution [31,32]. In sports and physical activity contexts, faster response times may contribute to more effective interactions with dynamic environmental demands, particularly during adolescence, a developmental period characterized by ongoing cognitive and motor maturation. Therefore, investigating how attentional focus strategies influence response-time performance may provide relevant insights into perceptual–motor functioning in youth populations. Thus, our goal is to explore the effects of EF and IF on adolescent motor performance. Participants under an IF are expected to perform worse than those in the control and EF groups. In addition, sports participation may be associated with differences in adolescents’ responses to attentional focus strategies during performance tasks. Finally, we aimed to examine whether a correlation exists between response time (best trial) and the number of hours adolescents spend participating in sports. Based on the existing literature, three main hypotheses were formulated: adolescents who received EF were expected to demonstrate significantly faster response times than those exposed to IF. We explored whether adolescents who engaged in regular sports practice would exhibit different response patterns under distinct attentional focus conditions, particularly under EF instructions. Finally, as a complementary exploratory analysis, we examined whether the number of weekly hours spent participating in sports was associated with response-time performance. It was expected that greater involvement in sports practice would be associated with faster responses, due to enhanced perceptual–motor efficiency developed through repeated practice.

## 2. Materials and Methods

### 2.1. Participants

A total of 58 participants (51.7% male, 48.3% female; mean age = 16.46 years) were recruited from three high school classes in a Portuguese school using a convenience sampling approach. Descriptive values of the sample are presented in Table 1. Regarding sex distribution, 21 males (58.3%) and 15 females (41.7%) participated in sports, while 9 males (40.9%) and 13 females (59.1%) were not engaged in any after-school sports practice. The inclusion criteria were being a high school student and not having any physical or cognitive impairments that would prevent testing (reports provided by parents). Participants who practiced sports regularly and those who did not were assigned to two separate groups. The sports-participation grouping referred specifically to adolescents engaged in organized sports practice outside regular school physical education classes. None of the participants had experience with the task.

### 2.2. Procedures and Tasks

All participants agreed to participate, and their legal guardians signed the consent form before testing. The University Ethics Committee approved the research (Protocol: UÉ24207), and all procedures followed the ethical guidelines of the Declaration of Helsinki [33]. Before the experimental protocol, all participants completed a demographic questionnaire that included their age, sex, and leg dominance. The experimental protocol included three groups: Control, Internal Focus of Attention (IF), and External Focus of Attention (EF), with participants randomly assigned to one of these groups. Six trials were completed, consisting of one familiarization trial followed by five test trials, with 20-s intervals. The best performance trial (lowest response time) was selected for analysis to represent each participant’s optimal perceptual–motor performance under the assigned attentional focus condition. All tests were conducted in a controlled environment, free from external noise or distractions that could affect participants’ attention. Additionally, they were naive about the experiment’s goals.

Before testing, all participants received the same general instruction: press the pods as quickly as possible when they are lit. Participants performed a familiarization trial, which was immediately followed by five trials to assess performance. Each group received specific information to direct their attention during practice. The attentional focus instructions were provided verbally before the test trials began and were not repeated during task execution. The Internal Focus of Attention group was instructed to ‘pay attention to the movement of your arms. In contrast, the External Focus of Attention group was told to ‘pay attention to the lights that turn on.’ The Control group did not receive any attentional focus instructions or feedback.

#### Response Time Task

**Figure 1:**
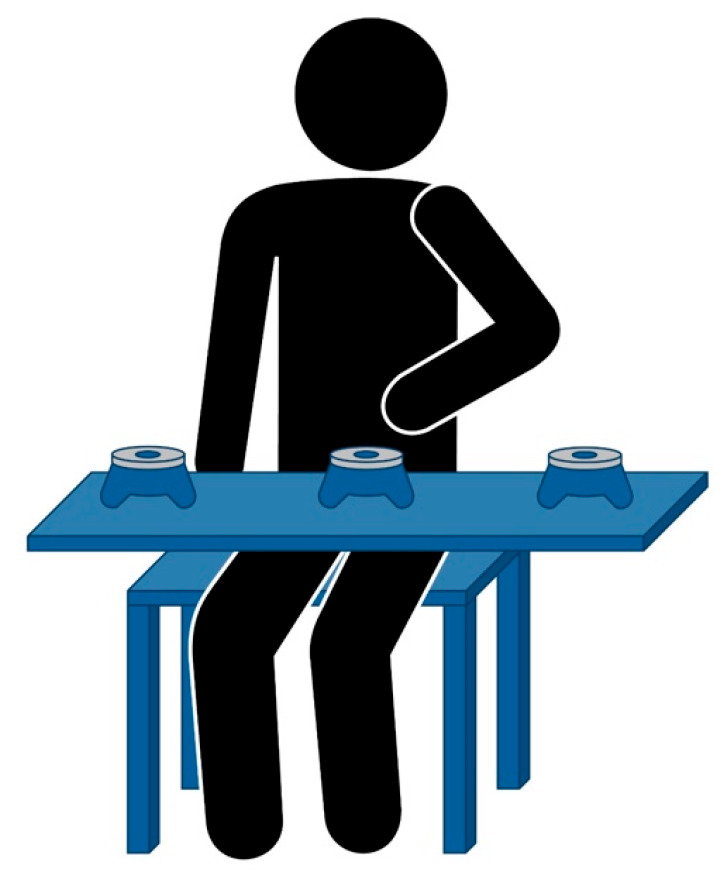
Description of the Response Time task. Note: Image provided by the authors.

The objective of the test was to measure the time interval, in milliseconds, between the presentation of a visual stimulus (pod light) and the participant’s response [34]. The test setup included three pods positioned in a row on a table, with 35 cm between each pod (see Figure 1) (BlazePod^TM^, Tel Aviv, Israel). Participants sat facing the central pod with their hands on the table to perform the task. The task always started with an auditory beep (controlled by the researcher), after which the pods lit up and turned off alternately at random intervals ranging from 0.5 to 1.5 s. Each response-time trial lasted 15 s, during which participants responded to as many visual stimuli as possible. Light stimulus remained active until the participant responded by pressing the corresponding pod. Consequently, participants with faster response times completed more stimuli within the same testing period.

### 2.3. Data Analysis

Descriptive statistics, with mean and standard deviation, were used to characterize the sample and summarize performance across the three experimental conditions. The Kolmogorov–Smirnov test confirmed that the dependent variable (response time) was normally distributed across groups (*p* > 0.05), and Levene’s test confirmed the homogeneity of variances (*p* > 0.05). To analyze the effects of attentional focus and sports practice on response time, a two-way factorial ANOVA (3 × 2) was conducted. Effect sizes for the ANOVA were reported using partial eta squared (η^2^p) and interpreted according to conventional benchmarks, with values of 0.01, 0.06, and 0.14 representing small, medium, and large effects, respectively [35]. Pearson correlation was used to examine the relationship between response time and the number of weekly hours of sports participation. Correlation coefficients were interpreted as small (r ≥ 0.10), medium (r ≥ 0.30), and large (r ≥ 0.50) effect sizes [35]. The between-subjects factors were attentional focus (internal, external, control) and sports practice (yes, no), with response time (measured in milliseconds) as the dependent variable. Data were analyzed using IBM SPSS Statistics for Windows, Version 29.0 (IBM Corp., Armonk, NY, USA) a significance level of 0.05.

## 3. Results

Table 2 presents the general results, controlled for group and sports practice. Descriptive analysis showed that participants in the EF group had the fastest response times overall, followed by the control group. Among participants, those who practiced sports had slightly faster response times than those who did not.

**Figure 2:**
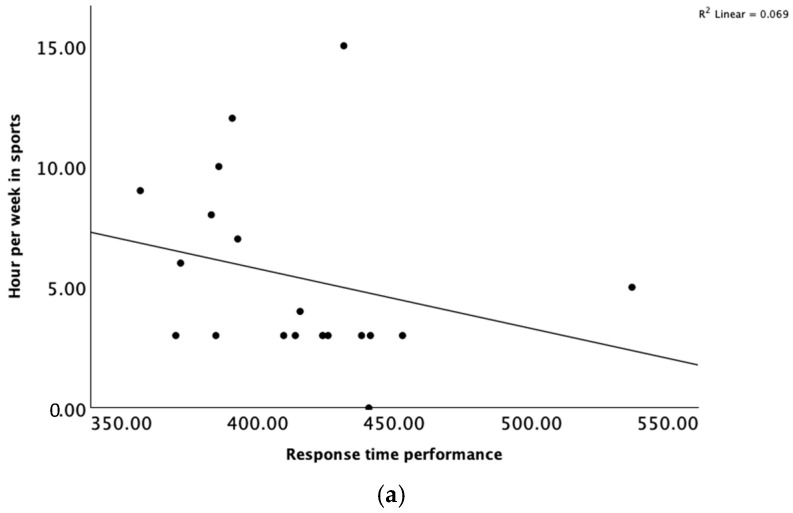

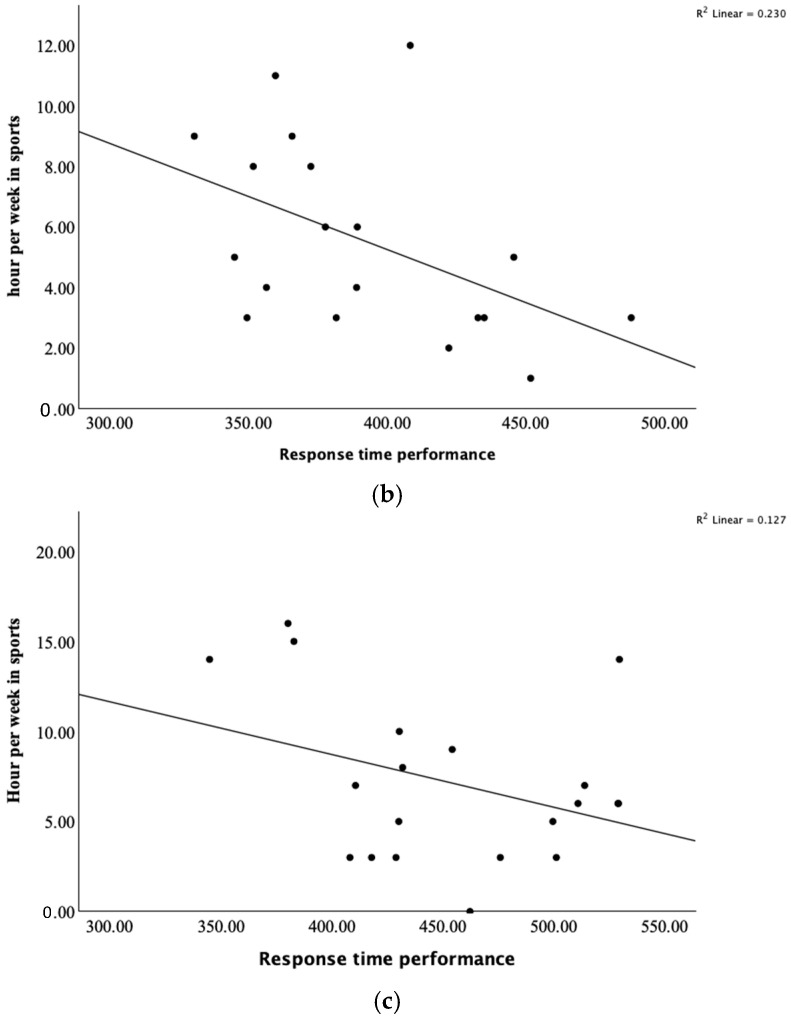
Associations between hours in sports participation and response time performance, controlling for focus groups. (**a**) Control group, (**b**) External focus of attention group, (**c**) Internal focus of attention group.

Correlation analysis revealed a moderately significant negative relationship between weekly hours of sports participation and response time in the EF group (r = −0.48, *p* = 0.04), indicating that greater sports participation was associated with faster response times (Figure 2). No significant associations between sports hours and performance were found in the control group (r = −0.26, *p* = 0.27) or in the IF group (r = −0.36, *p* = 0.12).

General analysis revealed a significant main effect of attentional focus group on response time (F(2, 52) = 6.18, *p* < 0.01, η^2^p = 0.19). Pairwise comparisons indicated that the IF group performed significantly worse than both the EF (*p* < 0.01, mean difference = 53.73 ms) and control (*p* = 0.02, mean difference = 38.09 ms) groups. The difference between control and EF was not significant (*p* = 0.31). The main effect of sports practice was not statistically significant (F(1, 52) = 2.11, *p* = 0.15, η^2^p = 0.04, suggesting that sports practice alone did not significantly affect response time.

**Figure 3:**
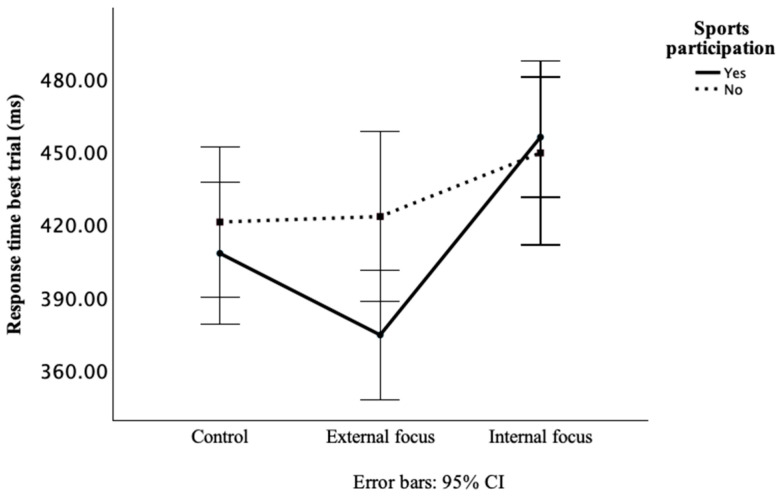
Comparisons between focus groups controlled by sports participation.

Although the interaction between attentional focus and sports practice was not statistically significant (F(2, 52) = 1.60, *p* = 0.21, η^2^p = 0.06), exploratory simple-effects analyses were conducted to further examine potential subgroup patterns and should therefore be interpreted cautiously. Among adolescents engaged in sports practice, significant differences were observed between attentional focus groups (F(2, 52) = 10.237, *p* < 0.01, η^2^p = 0.28). Post hoc analyses indicated that participants in the EF group demonstrated significantly faster response times than those in the IF group (*p* < 0.01), while the control group also outperformed the IF group (*p* = 0.02). In contrast, no significant differences were found between attentional focus conditions among non-sports participants (F(2, 52) = 0.78, *p* = 0.47). Additional pairwise comparisons indicated a significant difference between sports participants and non-participants only within the EF group (*p* = 0.03), with sports participants demonstrating better performance. No significant differences between sports participants and non-participants were observed in the control or IF groups (*p* > 0.05). See Figure 3 for further details.

## 4. Discussion

The present investigation analyzed how attentional focus influences response time performance in adolescents and explored whether sports participation was associated with different response patterns across attentional focus conditions. General findings align with previous literature, demonstrating a significant benefit of EF on response time performance. Exploratory subgroup analyses suggested that these effects may have been more pronounced among adolescents engaged in sports practice [12,19,36,37,38,39,40]. In contrast, IF consistently performed worse than the EF and control groups, particularly among sports participants. These results support the main hypothesis of this investigation and reinforce the principles of the Constrained Action Hypothesis [9,10], which proposes that EF facilitates more automatic and efficient motor control, an essential part of the information processing and response time [31,32], while IF promotes conscious control that interferes with performance. Although the interaction effect was not statistically significant, exploratory analyses suggested that adolescents who practiced sports regularly may have responded more favorably to EF instructions [19].

Despite growing empirical support for EF across ages and tasks [13,14,25], the current results address an existing gap by focusing on adolescent performance on response-time tasks requiring high-speed information processing. Our results are consistent with those of Marchant et al. [30] and Schwab et al. [29], who found that adolescents perform better with EF instructions in jumping and kicking tasks, respectively. On the other hand, Saemi et al. [19] noted no significant EF effect in beginner swimmers. It is also important to highlight that these findings challenge the current instructional practices commonly employed by coaches and physical education teachers, who often prioritize IF instructions and feedback during training and competition [1]. Interestingly, the authors also found a similar preference among athletes. This raises an important question: Is the performer’s preference for IF something that emerges naturally, or is it shaped over time by their teachers and coaches? As shown in the present results, although well-intentioned, this strategy (adopting IF) may inadvertently decrease performance. Therefore, the findings of this investigation suggest that EF instructions may positively influence performance on controlled perceptual–motor tasks. However, caution is warranted when extending these findings to more complex educational or sport-specific environments. In addition to the main effects of attentional focus, the correlation analysis offered supplementary insights into the relationship between sports participation and response time performance [41]. The significant negative association between weekly hours of sports participation and response time in the EF group suggests that adolescents who engage in sports more regularly may be more responsive to EF instruction/feedback. This finding aligns with the idea that motor experience enhances sensitivity to attentional processing [16], thereby enabling more efficient information processing and faster responses. Interestingly, this association was not observed in the control or IF groups, suggesting that prior sports experience may be associated with greater responsiveness to EF strategies. These findings may suggest that attentional focus and individual practice records should be considered in future investigations examining adolescent perceptual–motor performance.

While the present findings provide important insights into the effects of attentional focus on adolescent response-time performance, several limitations should be acknowledged. First, despite the experimental design, the relatively small sample size may limit the generalizability of the findings. In addition, the task consisted of a simple seated visual response-time paradigm performed under highly controlled conditions. Although this approach allowed greater experimental control and the isolation of perceptual–motor processes, it may not fully reflect the cognitive, coordinative, and contextual demands present in more complex or sport-specific environments, thereby limiting the ecological validity of the findings. Furthermore, the task required manual responses, which may not adequately represent the performance demands of lower-limb-dominant sports such as soccer or futsal. Another limitation concerns the analytical strategy, which relied on participants’ best-performance trial rather than on average performance across trials. While this approach enabled the identification of optimal perceptual–motor responses under each attentional focus condition, it may not fully capture performance consistency or within-participant variability throughout practice. Additionally, the cross-sectional design prevents drawing conclusions about long-term learning effects or the stability of attentional focus benefits over time. Finally, it was not possible to determine the type of attentional focus instructions or feedback to which participants had previously been exposed during sports practice or physical education classes. Future research should include larger, more diverse adolescent samples and a broader range of motor tasks that involve coordination, agility, decision-making, and sport-specific demands. Longitudinal and repeated-measures designs may also help clarify the long-term effects, stability, and practical applicability of attentional focus strategies across different performance contexts.

The present findings provide preliminary practical insights regarding the potential influence of attentional focus instructions on adolescent perceptual–motor performance. Specifically, the results suggest that external attentional cues may be associated with improved performance during controlled response-time tasks. However, given the highly controlled nature of the experimental task, caution is warranted when extending these findings to more complex educational, physical education, or sport-specific environments. Therefore, further investigations using ecologically valid and sport-specific tasks are necessary before broader practical recommendations can be established.

## 5. Conclusions

This study corroborates previous investigations and reinforces the importance of attentional focus in adolescent motor performance. An EF led to significantly better response times than IF or no-focus conditions, particularly among adolescents engaged in sports practice, although this finding emerged from exploratory analyses and should be interpreted with caution. While sports practice alone did not significantly improve performance, exploratory analyses suggested that adolescents engaged in sports practice may respond more favorably to EF strategies. However, this finding should also be interpreted cautiously, given the absence of a significant interaction effect. The findings provide preliminary support for the potential relevance of EF strategies when designing perceptual–motor activities for adolescents. However, further investigations using more complex and ecologically valid tasks are needed before broader practical recommendations can be established.

## Figures and Tables

**Table 1:** Characterization of the sample.

Group	Sports Participation	Variables	n	M	SD
Control	Yes (6 males; 4 females)	BMI (kg/m^2^)	10	22.20	3.17
		Age (years)	10	17.12	2.02
		Weekly sports participation hours **	10	7.90	3.73
		Hours playing video games *	10	1.60	1.35
	No (4 males; 5 females)	BMI (kg/m^2^)	9	22.22	2.68
		Age (years)	9	16.18	1.05
		Hours practicing physical education classes *	9	2.67	1.00
		Hours playing video games *	9	1.33	1.32
External focus of attention	Yes (7 males; 5 females)	BMI (kg/m^2^)	12	21.74	2.13
		Age (years)	12	16.68	1.33
		Weekly sports participation hours **	12	7.25	2.67
		Hours playing video games *	12	0.92	1.08
	No (3 males; 4 females)	BMI (kg/m^2^)	7	19.97	3.59
		Age (years)	7	15.47	1.27
		Hours practicing physical education classes *	7	2.57	0.79
		Hours playing video games *	7	1.00	1.15
Internal focus of attention	Yes (8 males; 6 females)	BMI (kg/m^2^)	14	21.84	2.63
		Age (years)	14	16.34	1.29
		Weekly sports participation hours **	14	9.14	3.96
		Hours playing video games *	14	1.57	1.09
	No (2 males; 4 females)	BMI (kg/m^2^)	6	24.26	4.50
		Age (years)	6	16.76	1.40
		Hours practicing physical education classes *	6	2.50	1.22
		Hours playing video games *	6	0.67	0.82

Note: * Mean hours per week; ** Weekly hours of sports participation besides school physical education classes.

**Table 2:** General results regarding the focus of attention and response time performance.

Group	Sports Participation	Trials	n	Mean Time (ms)	SD
Control	Yes	Familiarization	10	430.10	158.69
		T1	10	451.88	95.61
		T2	10	449.53	67.48
		T3	10	437.60	63.81
		T4	10	464.10	75.76
		T5	10	432.67	70.50
		Best performance	10	407.86	50.39
	No	Familiarization	9	550.86	71.09
		T1	9	464.58	44.93
		T2	9	434.81	32.98
		T3	9	468.22	66.57
		T4	9	467.75	47.28
		T5	9	454.37	47.40
		Best performance	9	420.73	27.82
External focus of attention	Yes	Familiarization	12	444.25	54.48
		T1	12	416.79	50.82
		T2	12	409.15	64.74
		T3	12	408.90	77.33
		T4	12	391.67	31.89
		T5	12	408.86	40.93
		Best performance	12	374.31	31.03
	No	Familiarization	7	525.86	72.75
		T1	7	462.96	68.94
		T2	7	434.57	43.79
		T3	7	442.18	41.72
		T4	7	447.21	45.15
		T5	7	452.33	53.04
		Best performance	7	422.99	45.46
Internal focus of attention	Yes	Familiarization	14	459.29	217.66
		T1	14	502.57	82.88
		T2	14	487.38	54.84
		T3	14	495.20	97.07
		T4	14	500.18	78.96
		T5	14	495.26	79.63
		Best performance	14	445.62	63.08
	No	Familiarization	6	470.67	234.31
		T1	6	498.38	50.42
		T2	6	521.08	94.64
		T3	6	482.42	56.62
		T4	6	486.88	66.43
		T5	6	478.22	31.86
		Best performance	6	449.14	36.49

## Data Availability

The data supporting the findings of this study are available from the corresponding author upon reasonable request. The participants did not provide written consent for public data sharing due to the sensitive nature of the research.
